# Purification-Driven Modulation of Polyphenol Profile and Protein Glycation-Inhibitory Potential of *Actinidia arguta* and *Actinidia kolomikta* Fruit Extracts

**DOI:** 10.3390/molecules31111935

**Published:** 2026-06-03

**Authors:** Artur Szwengiel, Tomasz Sawicki, Monika Jabłońska, Piotr Latocha, Wioletta Błaszczak

**Affiliations:** 1 Institute of Food Technology of Plant Origin, Faculty of Food Science and Nutrition, Poznań University of Life Sciences, ul. Wojska Polskiego 31, 60-624 Poznań, Poland; artur.szwengiel@up.poznan.pl; 2 Department of Human Nutrition, Faculty of Food Sciences, University of Warmia and Mazury in Olsztyn, ul. Słoneczna 45F, 10-719 Olsztyn, Poland; tomasz.sawicki@uwm.edu.pl (T.S.);; 3 Institute of Horticulture Sciences, Warsaw University of Life Sciences—SGGW, Nowoursynowska 166, 02-787 Warsaw, Poland; piotr_latocha@sggw.edu.pl; 4 InLife Institute of Animal Reproduction and Food Research, Polish Academy of Sciences, Trylińskiego 18, 10-748 Olsztyn, Poland

**Keywords:** *Actinidia* fruit, extract purification, polyphenols profiling, antioxidant capacity, antiglycaemic potential

## Abstract

This study characterises the polyphenol profile (LC–MS), antiglycation (BSA–GLU, BSA–FRU, BSA–MGO), and antioxidant potential (ABTS, DPPH) of *A. arguta* (‘Scarlet September Kiwi’) and *A. kolomikta* (‘Lande’) fruit before and after extract purification. A total of 48 polyphenols belonging to 11 chemical groups were identified. Crude Scarlet extract showed higher TPI (1079.51 µg/g dw) than the crude Lande (761.13 µg/g dw), with quercetin glucoside accounting for 71% of TPI. Crude Lande extract was dominated by caffeic acid glucoside (39% TPI). Purification markedly increased TPI values to 4082.13 µg/g dw (Scarlet) and 2550.51 µg/g dw (Lande). The crude extracts more effectively inhibited glucose- and fructose-induced protein glycation (IC_50_ = 1.81–7.71 mg/mL) than methylglyoxal-mediated glycation (IC_50_ = 14.33–24.26). Purification significantly enhanced antiglycation efficacy in all models (IC_50_ = 0.47–1.78), as well as antioxidant capacity (IC_50_ = 0.10–0.67 mg/mL). Statistical analyses revealed a strong alignment between the glycation-inhibitory activity and the tested antioxidant potential. These findings suggest that targeted purification enhances the functional potential of *Actinidia* species, making them promising sources of bioactive compounds against oxidative stress and protein glycation-related disorders.

## 1. Introduction

Recently, the number of research articles concerning the fruit of *Actinidia arguta* Miq. (baby kiwi) and *Actinidia kolomikta* Maxim (arctic kiwi) has increased substantially, providing comprehensive insights into the profile of antioxidants and their impact on the health-promoting effects in vitro and in vivo [1,2,3,4]. As shown, *A. arguta* fruit is characterised by a high concentration of ellagic acid (557.00 µg/g dw), neochlorogenic acid (142.10 µg/g dw), kaempferol-3-O-galactoside (152.20 µg/g dw) or rutin (146.48 µg/g dw) [2,4]. In contrast, the fruit of *A. kolomikta* had a high content of L-ascorbic acid (8271.96 µg/g dw), (+)-catechin (827.17 µg/g dw), procyanidin C1 (1236.66 µg/g dw), and chlorogenic acid (125.92 µg/g dw). Similarly, caffeic acid (323.71 µg/g dw), ferulic acid (322.33 µg/g dw) and quercetin (54.10 µg/g dw) were found at relatively high levels in the fruit of both *Actinidia* species [2,3]. Actinidia polyphenols may exert a range of protective mechanisms, including antioxidant, anti-inflammatory, and protein glycation-inhibitory effects. The latter is particularly relevant, as the formation of advanced glycation end-products (AGEs) is associated with various health disorders, including chronic oxidative stress and type 2 diabetes [5,6].

AGEs are a heterogeneous group of compounds formed through non-enzymatic glycation reactions between reducing sugars and free amino groups of proteins. They can arise endogenously during natural ageing or under pathological conditions such as inflammation and hyperglycaemia. Dietary AGEs, on the other hand, are formed during high-temperature food processing and are also promoted by diets rich in fructose-containing sweeteners [7,8]. Although fruits provide natural fructose together with antioxidants that may counteract glycation processes, excessive intake of processed fructose sources such as sucrose and high-fructose corn syrup may overwhelm these protective mechanisms. Both exogenous and endogenous AGEs exert similar detrimental effects on biological functions [9,10]. Under pathological conditions, oxidative stress enhances the reactivity of reducing sugars towards proteins, accelerating the formation of reactive intermediates such as methylglyoxal (MGO) and glyoxal (GO), ultimately leading to irreversible AGE formation [11]. Therefore, scavenging reactive oxygen species and trapping MGO or GO using dietary polyphenols has been widely proposed as an effective strategy to inhibit protein glycation *in vitro* [12].

Importantly, in complex food matrices, polyphenols may interact with polysaccharides, proteins, and organic acids through non-covalent interactions, including hydrogen bonding, hydrophobic forces, electrostatic interactions, and π–π stacking. These interactions can significantly influence the solubility, stability, and bioavailability of polyphenols, thereby modulating their biological activity [13,14]. At the same time, such matrix interactions may mask or reduce the apparent bioactivity of phenolic compounds in crude extracts.

To address this limitation and improve the interpretability of biological effects, purification strategies that remove non-phenolic matrix components (sugars, organic acids, pectins, and proteins) can be applied, thereby allowing a more direct assessment of polyphenol-driven activity.

Therefore, in the present study, both crude and purified extracts from *A. arguta* (cv. ‘Scarlet September Kiwi’) and *A. kolomikta* (cv. ‘Lande’) fruit were investigated. The purified extracts were obtained via removal of non-phenolic constituents, enabling a direct evaluation of how matrix components influence anti-AGE activity. This comparative approach represents the main novelty of the study, as it allows discrimination between the activity of crude fruit extracts and their polyphenol-enriched fractions, which has not been sufficiently explored for *Actinidia* species. To investigate this, we employed the well-established BSA–glucose and BSA–MGO in vitro models, commonly used to evaluate anti-AGE effects of plant-derived compounds [15]. The BSA–FRU model was also applied to assess extract efficacy against fructose-mediated protein glycation *in vitro* [16]. Additionally, targeted and untargeted LC–MS/MS analyses were combined to comprehensively characterise polyphenols in the obtained extracts, thereby linking the profile of polyphenols with observed bioactivity.

## 2. Result and Discussion

### 2.1. Polyphenol Profiling

In this study, *Actinidia arguta* (*cv.* ‘Scarlet September Kiwi’) and *Actinidia kolomikta* (*cv.* ‘Lande’) are referred to as ‘Scarlet’ and ‘Lande’, respectively, throughout the text and tables.

A total of 48 polyphenols were identified in the ‘Scarlet September Kiwi’ and ‘Lande’ fruit extracts using both reference standards and an untargeted approach, and the identified compounds were classified into 11 groups based on the ClassyFire algorithm (Table 1 and Table S1).

To evaluate the reproducibility of the purification procedure and subsequent LC-MS/MS analyses, the results obtained for purified extracts (each representing a pooled pair from six independent extract replicates) are presented separately in Table 2, consistent with the records in Table S1. This presentation allows a clear assessment of variability across independent preparations, providing confidence in the reliability of the identified compounds before proceeding to comparative *in vitro* analyses. 

As shown in Table 1, the number of compounds increased after purification of the crude extracts, with most compounds belonging to the group of flavonoid-O-glycosides, biflavonoids and polyflavonoids, and phenolic glycosides. A comparison of the crude and purified extracts shows that the increase in the number of compounds within particular chemical classes results from certain compounds being below the detection limit in the crude samples (Table 2). Our results are consistent with those of Ćesoniene and co-authors [3], who also found flavan-3-ols, flavonols, phenolic acids, and flavones in Lande fruit (crude extract). According to these authors, the total phenolic compound content in the tested fruit was 985.15 µg/g, which is approximately 30% higher than that obtained in our study. On the other hand, *A. arguta* fruit cultivated in China (‘Longcheng No. 2’), in addition to flavanols, flavonols, and phenolic acids, also serves as a source of anthocyanins [17].

Data presented in Table 2 indicate that the crude Scarlet extract exhibited an approximately 42% higher TPI value (sum of individual phenolics concentration) compared to the crude Lande extract. Moreover, a total of 28 individual polyphenols were detected in the crude Scarlet extract, whereas the Lande crude material contained seven fewer compounds. The Mann–Whitney U test showed significant differences in the medians (*p* < 0.05) between the Lande and Scarlet crude extracts for all tested compounds. Similar results were obtained for the purified extracts, except for the concentration of coumaric acid glucoside, p-coumaric acid, and the flavonoid kaempferol glucoside (*p* > 0.05). Among the identified polyphenols, quercetin glucoside was the predominant compound in the crude Scarlet extract, accounting for 71% of the TPI, whereas caffeic acid glucoside, identified at *RT* = 6.52 (Table S1), followed by epicatechin and procyanidin B2, were the major compounds in the crude Lande extract, representing 39%, 17% and 13% of the TPI, respectively (Table 2). Our findings are in line with those of Ćesoniene and co-authors [5], who noted that, among the tested polyphenols, (-)-epicatechin (200.45–456.71 µg/g dw) and/or procyanidin B2 (157.52–465.27 µg/g dw) dominated in the majority of analysed *A. kolomikta* fruit *cvs* grown in Lithuania. However, according to these authors, the dominant polyphenol group identified in *A. arguta* fruit was flavonols, with isoquercitrin ranging from 257.02 to 621.23 µg/g dw, whereas it was found that hydroxybenzoic acids (21.47 µg/g dw) and hydroxycinnamic acids (15.21 µg/g dw) with hydroxybenzoic acid hexoside (13.42 µg/g dw) and caffeoyl hexoside (5.74 µg/g dw), respectively, accounted for the majority of non-anthocyanin polyphenols in the tested *A. arguta* fruit [17].

An average three-fold increase in quercetin glucoside concentration was observed in the purified Scarlet extracts, reaching 57% of the TPI. Moreover, a nearby seven-fold increase was observed for rutin and neochlorogenic/cryptochlorogenic acid content in the purified Scarlet extracts, accounting for average levels of 9% and 7% of TPI, respectively. As shown in Table 2, procyanidin and procyanidin B2 were also among the main polyphenols demonstrating seven-fold and six-fold increases, respectively, after the crude extracts purification. Despite that distinct increase, the concentration of the above-mentioned phenolics accounted for about 4% (on average) of the TPI. Alike, the concentrations of epicatechin and catechin increased six-fold and five-fold, respectively, after purification of the crude Scarlet extracts, representing an average of about 3% of TPI. A total of sixteen new polyphenols were detected in the purified Scarlet extracts. However, their contribution to the TPI was relatively low, ranging from 0.01% (caffeic acid) to 0.23% (proanthocyanidin).

As shown by the data in Table 2, the purified Lande extracts had a three-fold higher (on average) TPI compared to the crude material. A twofold, fourfold, and sixfold increase in the concentration of caffeic acid glucoside (*RT* = 6.52), epicatechin, and procyanidin B2, respectively, was observed after purification of the crude Lande extracts. These phenolics remained the dominant compounds in the purified extract, accounting for 23%, 19%, and 21% of the TPI value, respectively. Moreover, the purified Lande extracts exhibited fivefold and fourfold higher concentrations of quercetin glucoside and kaempferol glucoside, respectively, compared to the crude material, with these compounds accounting for approximately 11% and 8% of the TPI. Purification of the crude Lande extracts induced a fourfold and fivefold increase in the concentration of catechin and procyanidin. We also found that the purified Lande extracts had a threefold higher concentration of caffeic acid glucoside, identified at *RT* = 4.98 (Table S1). These polyphenols represented about 3% of TPI. A total of twelve new polyphenols were noted as a result of the purification of the crude Lande extracts (Table 2). Among these compounds, the lowest contribution (0.01%) to TPI was found for caffeic acid, naringenin, and flavonoid coumaroyl glycoside, whereas proanthocyanidins (*RT* = 7.85 and *RT* = 9.11) constituted around 1% of the TPI value. The profiling of polyphenols proved that the tested *Actinidia* genotype strongly determined the presence of some individual polyphenols. For example, gallocatechin, epigallocatechin, proanthocyanidin, feruloyl hexoside, feruloylquinic acid, myricetin glucopyranoside, quercetin rhamnoside, quercetin-3-O-glucosyl-6″-acetate, and a feruloylated flavonoid glucoside were detected exclusively in the Scarlet (*A. arguta*) fruit. Conversely, caffeic acid glucoside, chlorogenic acid, and a flavonoid glycoside were found only in the Lande (*A. kolomikta*) fruit.

It should be noted that the increased concentrations of polyphenols observed after purification most likely resulted from matrix reduction rather than from an absolute increase in phenolic compounds. The removal of non-phenolic constituents, including sugars, organic acids, proteins, and pectins, decreased the total extract mass and consequently increased the relative proportion of phenolic compounds in the purified fractions. Similar enrichment effects after adsorption-based purification have previously been described for polyphenol-rich plant matrices [18]. Furthermore, some phenolic compounds detected in the purified fractions may have already been present in the crude extracts at low concentrations but remained less detectable due to matrix interference caused by abundant non-phenolic constituents. The reduction in matrix complexity after purification likely enhanced both the chromatographic separation and analytical detectability of these minor phenolic compounds.

### 2.2. Anti-AGEs Potential

The anti-AGE potential of the analysed extracts from actinidia fruit was quantified using IC_50_ values, indicating the extract concentration (mg/mL) that resulted in 50% inhibition of AGE formation (Table 3).

As shown in Table 3, the tested crude extracts exhibited a distinctly higher protective effect against albumin damage induced by GLU and FRU (IC_50_= 1.81–7.71) than by MGO (IC_50_ = 14.33–24.26). The obtained results also indicated that the crude Scarlet extracts showed higher inhibitory potential on AGEs formation than the crude Lande extracts. As reported in the literature, non-enzymatic glycation begins with the formation of a reversible Schiff base between a reducing sugar and a protein amino group. Aldoses such as GLU rearrange into Amadori products, whereas ketoses such as FRU form Heyns products. These early glycation products can undergo further rearrangements into a variety of highly reactive dicarbonyls, which next participate in a complex of parallel, sequential, and branched chains of irreversible nonenzymatic reactions, ultimately producing a variety of AGEs [8,10,19]. As noted, polyphenols such as chlorogenic acid, epigallocatechin gallate and quercetin are potent inhibitors of α-dicarbonyl compounds [8]. However, it should be noted that flavonoids may differ in their antiglycation effects depending on the glycation model used [20]. According to the above-cited reference, a proanthocyanidin-rich fraction from fruit peel and quercetin exhibited strong inhibitory effects on AGEs formation (IC_50_ = 7.9 and 15.6 µg/mL, respectively) at the early stage of glycation, as evaluated using the BSA–FRU model. Moreover, it was also found that in dicarbonyl-mediated glycation models (BSA–MGO and ARG–MGO), quercetin showed markedly lower IC_50_ values (24.4 and 70.3 µg/mL, respectively) than the proanthocyanidin-rich fraction (128.9 and 249.3 µg/mL, respectively), indicating a higher efficiency of quercetin in scavenging reactive carbonyl intermediates such as MGO [20]. Consistent with these findings, our study demonstrated that the crude Scarlet extract contained distinctly higher concentrations of quercetin, quercetin glucoside and other quercetin derivatives compared to the crude Lande extract. Interestingly, among the tested crude extracts, only the crude Scarlet extract showed the presence of a proanthocyanidin compound (RT = 6.05) (Table 1 and Table S1). This compositional difference may account for the nearly two-fold stronger protective effect of the crude Scarlet extract against MGO-induced BSA damage relative to the Lande extract. Moreover, our previous study also indicated pronounced antiglycation activity of Scarlet fruit relative to Lande, particularly in the context of MGO-mediated protein damage (IC50 = 18.06 and 26.42 mg/mL, respectively) [2]. The antidiabetic potential of A. arguta fruit was supported in the literature by its inhibitory activity against α-amylase (IC_50_ = 4.13–6.62 µg/mL) and α-glucosidase (IC_50_ = 0.18–10.00 µg/mL) [6].

Moreover, data obtained in our work showed that the purification of the extracts distinctly increased the anti-AGEs potential of the tested fruit extracts across all in vitro models used (IC_50_ = 0.47–1.78) (Table 3). This finding is consistent with the understanding that the composition of the plant extract matrix can influence the inhibitory effects of polyphenols on AGEs formation [21]. BSA–glucose/fructose in vitro models are sensitive to the extract matrix, as all extract constituents may influence both the course of the glycation reaction and the measurement of early and late glycation products (e.g., fructosamine, fluorescent AGEs, and protein-derived products) [22]. Our findings are consistent with the aforementioned studies, indicating that the superior anti-AGEs protective activity of the purified Scarlet and Lande extracts results from the production of high-quality extracts characterised by high polyphenol concentrations (Table 2) and reduced levels of interfering non-phenolic compounds, compared to the corresponding crude extracts. Therefore, these observations highlight not only the importance of the polyphenol profile in determining antiglycation efficacy but also demonstrate that the presence of non-phenolic compounds in the fruit matrix appears to be a key factor influencing its antiglycation activity.

The practical relevance of the obtained IC_50_ values should also be considered in the context of commonly used antiglycation reference compounds. Aminoguanidine is widely applied as a standard inhibitor in in vitro antiglycation studies due to its strong carbonyl-trapping activity. However, despite its high efficacy, the clinical application of aminoguanidine has been limited because of its adverse side effects. Therefore, increasing attention has been directed towards naturally occurring dietary polyphenols as safer antiglycation agents. Although the purified actinidia extracts exhibited lower antiglycation potency than aminoguanidine, their inhibitory activity remained within the range reported for various polyphenol-rich plant extracts evaluated in comparable in vitro models [2,18]. Moreover, our previous study proved that actinidia extracts were characterised by even higher antiglycation potential than commercial phenolic standards [23]. Considering all the above, the IC_50_ values observed for the purified extracts (Table 3), particularly in the BSA–GLU and BSA–FRU models, indicate a biologically relevant antiglycation potential and support the possible application of actinidia fruit as a natural dietary source of antiglycation compounds.

### 2.3. Antioxidant Potential

ABTS and DPPH assays were employed to compare the antioxidant potential of the tested crude and purified actinidia extracts (Table 3). The obtained results demonstrated that crude Lande extracts exhibited a significantly higher antiradical capacity than crude Scarlet extracts. These findings are consistent with our previous studies, in which a comparative analysis of different *A. arguta* cultivars (Weiki and Scarlet September Kiwi) and the *A. kolomikta* genotype (Lande) revealed markedly stronger antioxidant properties for the Lande fruit extract (282.01–312.42 μmol Trolox/g dw) compared to Scarlet (20.46–76.67 μmol Trolox/g dw) [2]. Furthermore, literature data corroborate the high antiradical potential of *A. kolomikta* fruit, which, depending on the cultivar, may reach approximately 300–500 μmol Trolox/g dw [3]. Slightly higher IC_50_ values for *A. arguta* extracts obtained by ultrasound-assisted extraction (0.168 and 2.153 for FRAP and ABTS, respectively) were reported in another study [1], where a water–ethanol mixture (1:2.33, *v*/*v*) was used, potentially influencing the observed activity.

As expected, purification markedly enhanced the antiradical potential of the tested extracts (Table 3). Lande maintained high antioxidant activity, as assayed by ABTS, whereas no significant differences were observed between the tested purified extracts in the DPPH assay. Zeng and co-authors [24] investigated the in vitro and in vivo antioxidant activities using DPPH and ABTS of six structurally related flavonoids, including, among others, procyanidin B2, epicatechin, epigallocatechin, quercetin, and rutin. Among the tested compounds, procyanidin B2 exhibited the strongest in vitro radical-scavenging activity compared to epicatechin, quercetin, or rutin. The authors suggested that the number of phenolic hydroxyl groups and structural features, such as additional hydroxyls and double bonds, enhance antioxidant activity. Our results are consistent with these reports, showing that higher concentrations of polyphenols, including procyanidin B2, catechin, epicatechin and quercetin and/or quercetin derivatives, may contribute to the greater antiradical potential of the purified extracts compared to the crude extracts.

### 2.4. Statistical Analysis of Experimental Results

Exploratory data analysis was conducted, and dendrograms were generated using 5045 features extracted from MS spectra in positive mode (Figure S1A) and 509 features extracted from MS spectra in negative mode (Figure S1B). The two obtained patterns are consistent, although in positive mode, about 10 times more features were detected. The crude extracts form one cluster; however, after purification, the samples display pronounced genotype-dependent dissimilarity, driven by differences in their polyphenol profile. To facilitate an integrated, exploratory assessment of all variables and cases, a clustered heatmap was generated, as presented in Figure S2. The samples exhibit a similar pattern to that produced using all features extracted from MS spectra. The distinct structure also shows compounds, forming two groups on the heatmap. Certain compounds are particularly prevalent in the pure Scarlet group, while they exist at low concentrations in the second group. A comparable trend can also be observed in the pure Lande group. Before purification, the Scarlet and Lande extracts exhibited distinctly lower concentrations of all detected polyphenols.

**Figure 1:**
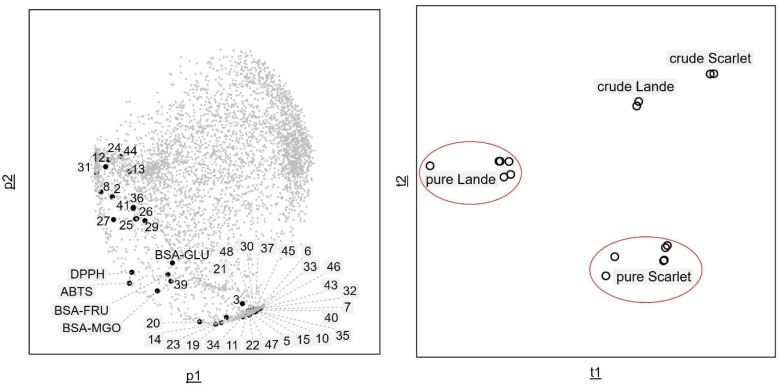
PLS—raw loadings (**left**) and scores (**right**) plots generated from LC-MS data in positive ion mode. The loadings indicate individual chemical compounds (**1**–**48**), antioxidant activity (ABTS, DPPH), and variables related to protein glycation inhibitory potential, calculated as 100 minus IC50. The score plot illustrates how the samples cluster.

**Figure 2:**
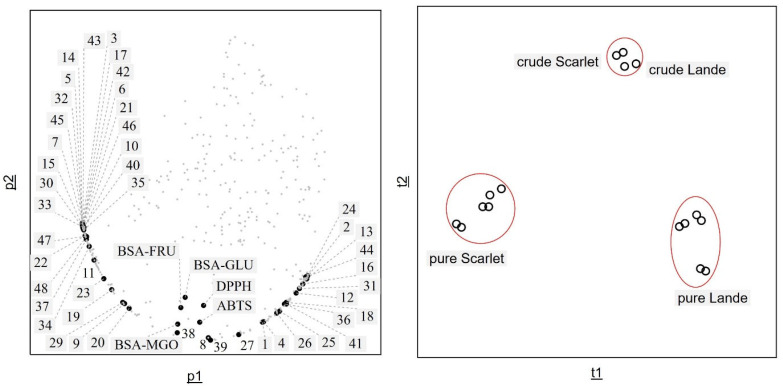
PLS—raw loadings (**left**) and scores (**right**) plots generated from LC-MS data in negative ion mode. The loadings indicate individual chemical compounds (**1**–**48**), antioxidant activity (ABTS, DPPH) and variables related to protein glycation inhibitory potential, calculated as 100 minus IC50. The score plot illustrates how the samples cluster.

Figure 1 and Figure 2 show the Partial Least Squares (PLS) model using paired loadings and score plots. The models were derived from LC-MS data acquired in positive and negative ionisation modes (untargeted approach) and from variables related to the samples’ protein glycation-inhibitory potential.

The PLS model shown in Figure 1 has two components that account for 67.3% of the variance in the predictor matrix (R^2^X) and 69.2% of the variance in the response matrix (R^2^Y). These components demonstrate acceptable predictive ability after cross-validation with a Q^2^ of 0.56. Analogically, the PLS model presented in Figure 2 has R^2^X = 0.47, R^2^Y = 0.68, and Q^2^ = 0.49.

The loadings plot (left panels) shows how variables are mapped onto the first two latent variables (p1 and p2). Compounds farther from the origin have a greater influence on the latent structure. Several variables align in the same direction, indicating positive relationships among them. The scores plot (right panels) illustrates the projection of samples onto the first two latent variables (t1 and t2), clearly showing clustering by sample type. There is a distinct separation between pure Scarlet and pure Lande samples, with each forming tight clusters reflecting consistent metabolomic profiles within its group. Crude extracts (crude Scarlet and crude Lande) are located separately from their purified fractions and from each other, indicating compositional differences, especially as seen in the model in Figure 1. The differences between crude samples are less apparent in Figure 2, which shows LC-MS data acquired in negative ion mode. Joint analysis of the scores and loadings plots reveals that the differentiation between sample groups is influenced by specific subsets of LC-MS compounds. Samples mapped to specific loading clusters exhibit higher levels of these metabolites, indicating candidate compounds that drive the observed chemical and functional differences.

The protein glycation-inhibitory potential variables and antioxidant activity indicators mapped onto the score plot fall between the pure Lande and Scarlet groups, suggesting that these samples do not exhibit unique chemical compounds associated with these variables. However, PLS results clearly indicate that the chemical profiles of these samples differ, and purification further accentuates this effect. Additionally, the direction of glycation-inhibitory vectors aligns with the samples’ antioxidant activity. Crude samples showed significantly lower values of the measured parameters, which explains why the vectors are in the opposite direction from their positions. These results distinctly suggest that glycation-inhibitory parameters are strongly associated with the increased antioxidant activity observed after extract purification. That finding is in line with the literature data indicating that polyphenols can inhibit protein glycation, primarily due to their antioxidant potential, including scavenging free radicals, chelating metal ions, and preventing secondary structure transitions in proteins [17].

**Figure 3:**
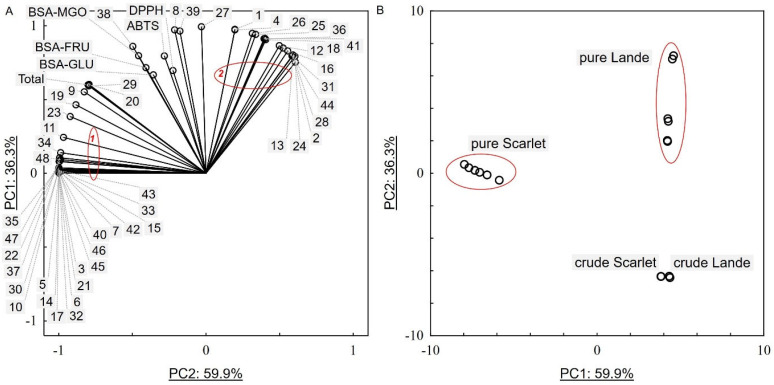
PCA ((**A**) correlation loading and (**B**) score plot) generated from 48 compounds identified in the MS spectra of crude and purified extracts, with variables representing the half-maximal inhibitory concentration (IC_50_) of these extracts from *A. arguta* (*cv.* Scarlet September Kiwi) and *A. kolomikta* (*cv.* Lande), expressed as 100 minus IC50 (protein glycation inhibitory potential: BSA-GLU, BSA-FRU, BSA-MGO; antioxidant potential: ABTS and DPPH). **1**—Salicylic acid glucoside, **2**—caffeic acid glucoside, **3**—quercetin vicianoside, **4**—dihydroxybenzoate glucoside, **5**—gallocatechin, **6**—proanthocyanidin, **7**—flavonoid glycoside (coumaric acid glycoside), **8**—coumaric acid glucoside, **9**—3,4–dihydroxybenzoic acid, **10**—neochlorogenic acid/cryptochlorogenic acid, **11**—flavonoid (taxifolin glucoside), **12**—fraxin/isofraxoside, **13**—caffeic acid glucoside, **14**—epigallocatechin, **15**—proanthocyanidin, **16**—sinapoulhexoside, **17**—feruloyl hexoside, **18**—coumaric acid glucoside, **19**—procyanidin, **20**—flavonoid, **21**—feruloylquinic acid, **22**—coumaroyl quinic acid, **23**—catechin, **24**—chlorogenic acid, **25**—epicatechin, **26**—procyanidin B2, **27**—proanthocyanidin, **28**—4–hydroxybenzoic acid, **29**—caffeic acid, **30**—quercetin–diglucoside, **31**—flavonoid, **32**—flavonoid glycoside (myricetin glucopyranoside), **33**—quercetin glycoside (quercetin rhamninoside), **34**—naringenin, **35**—rutin, **36**—proanthocyanidin, **37**—quercetin glucoside, **38**—p–coumaric acid, **39**—flavonoid (kaempferol glucoside), **40**—quercetin xyloside, **41**—kaempferol glucoside, **42**—quercetin–3–O–glucosyl–6″–acetate, **43**—quercetin malonylglucoside, **44**—flavonoid glycoside, **45**—flavonoid ferulylglucoside, **46**—flavonoid ferulylglucoside, **47**—flavonoid coumaroyl glycoside, **48**—quercetin.

PCA analysis was performed to show the relationships among polyphenolic compounds, protein glycation inhibition, and the antioxidant potential of extracts from Lande and Scarlet cultivars (Figure 3). The two principal components explain 96% of the variability in the results matrix. The results are consistent with the previously described heatmap and PLS models. However, it is evident that variables BSA-GLU, BSA-FRA, BSA-MGO, ABTS, and DPPH are correlated. The first set of vectors is highly abundant in the purified extract of cultivar Scarlet; the second set relates to the pure Lande samples. The crude extracts were not separated due to the low abundance of polyphenolic compounds, and their protein-glycation-inhibitory antioxidant potential was lower than that of their purified counterparts. Although some compounds (**8**—coumaric acid glucoside, **27**—proanthocyanidin, **38**—p–coumaric acid, **39**—flavonoid (Kaempferol glucoside)) are highly correlated with the BSA-GLU, BSA-FRA, BSA-MGO, ABTS, and DPPH vectors, they generally fall between two clusters of vectors, suggesting that the observed low IC_50_ values cannot be attributed to individual molecules, but rather arise from synergistic interactions within the polyphenolic matrix, linking the antioxidant and anti-AGEs activities of the tested actinidia fruit extracts.

## 3. Materials and Methods

### 3.1. Chemicals and Reagents

All standards, reagents, and solvents used in this research were of HPLC grade. The following reagents were obtained from Sigma Chemical Co. (Poznań, Poland): glucose (GLU), fructose (FRU), methylglyoxal (MGO), bovine serum albumin (BSA), aminoguanidine hydrochloride, phosphate-buffered saline (PBS), 2,2′-azinobis(3-ethylbenzothiazoline-6-sulfonic acid) diammonium salt (ABTS^•+^), 2,2-di(4-tert-octylphenyl)-1-picrylhydrazyl (DPPH), and 6-hydroxy-2,5,7,8-tetramethylchroman-2-carboxylic acid (Trolox). The remaining reagents (all of reagent grade quality) were supplied by POCh (Gliwice, Poland).

Reference standards used for LC–MS calibration were purchased from Sigma-Aldrich (Merck, Darmstadt, Germany) and Extrasynthese (Genay, France): 3,4-dihydroxybenzoic acid (≥97%), 4-hydroxybenzoic acid (≥99%), apigenin (≥95%), caffeic acid (≥98%), ferulic acid (≥98%), p-coumaric acid (≥98%), procyanidin B2 (≥97%), and sinapic acid (≥98%) from Sigma-Aldrich; catechin (≥99%), chlorogenic acid (≥99%), epicatechin (≥99%), epigallocatechin (≥98%), gallocatechin (≥98%), quercetin (≥99%), and rutin (≥99%) from Extrasynthese.

### 3.2. Actinidia Fruit

A total of two *Actinidia* genotypes cultivated in Poland were analysed in this study: *A. arguta* (*cv.* ‘Scarlet September Kiwi’) and *A. kolomikta* (*cv.* ‘Lande’). ‘Scarlet September Kiwi’ fruit was harvested from a commercial orchard (Minikiwi Kostrzewa, Bodzew, Belsk Duży, Poland), while ‘Lande’ fruit was collected from the Experimental Garden of the Institute of Horticultural Sciences, Warsaw University of Life Sciences (SGGW), Poland. Next, the fruit was transported to the InLife Institute of Animal Reproduction and Food Research, Polish Academy of Sciences, and stored in a cold chamber at 6 ± 2 °C until analysis (for no longer than 10 days).

Before analysis, the fruit was characterised for titratable acidity and soluble solids content according to ISO standards (ISO 750:1981 E [25] and ISO 2173:2003 [26], respectively). The measured values were as follows: 1.54 g/100 g citric acid and 16 °Brix for ‘Scarlet September Kiwi’ and 1.90 g/100 g citric acid and 14.5 °Brix for ‘Lande’. All analyses were performed in triplicate.

#### Fruit Sample Preparation

Whole fruits, including the peel, flesh, and seeds, were homogenised and subsequently lyophilised prior to extraction. Approximately 800 g of fruit was homogenised (9000 rpm/15 s) in three batches using a homogeniser (B-400, Buchi, Flawil, Switzerland). The homogenates were then frozen using liquid nitrogen and lyophilised. The obtained lyophilised material was placed in oxygen barrier bags, vacuum packed (Kubo 3048, Elegen, Scandiano, Italy), and stored at −24 °C until analysis.

### 3.3. Polyphenol Extraction

#### 3.3.1. Crude Extract

To obtain crude extracts, the polyphenol fraction from lyophilised material was extracted without exposure to high temperature using dynamic sonication (A.G.A. Analytical, Warsaw, Poland). The extraction mixture consisted of methanol and water (4:1, *v*/*v*). The extraction procedure was as follows: approximately 600 mg of the actinidia material was suspended in the extraction mixture (2 mL), and it was vortexed (3 × 60 s), sonicated (20 kHz/8 ± 2 °C/3 × 30 s) and centrifuged (14,000 rpm/4 °C/10 min). Each of the above-mentioned extraction steps was repeated five times. The supernatants were collected, and the methanol was then evaporated under a stream of nitrogen, and the remaining aqueous phase was removed by lyophilisation.

The extraction was carried out in six independent replicates for each *Actinidia* genotype. The average extraction yield of polyphenol crude extracts from Scarlet and Lande fruit was 65%.

#### 3.3.2. Crude Extract Purification

Purification of the crude extract was carried out according to the method described by Sanches-Gonzalez and co-authors [27], with modifications adapted to purify crude extract from actinidia fruit. Amberlite XAD-7HP (20–60 mesh, Sigma-Aldrich) was used, since this stationary phase allows for removing any protein, mucilage, pectin and other sugars from the crude extract. A glass column (32 cm × 1.5 cm) was filled with Amberlite XAD-7HP previously activated with double-distilled and degassed water (ddH_2_O).

From six independent replicates of crude extract obtained for each Actinidia genotype, every two were combined and applied to the column after being dissolved in 1.5 mL of methanol–water mixture (4:1, *v*/*v*). Any visible solid particles in the solution were removed by centrifugation (8000 rpm, 4 min) (MiniSpin plus, Eppendorf, Hamburg, Germany). High-molecular-weight compounds were eluted with an excess of double-distilled water (ddH_2_O), whereas the phenolic compounds adsorbed onto the resin were subsequently eluted with methanol until no UV-absorbing compounds (λ = 250–355 nm) characteristic of phenolics were detected in the eluate [28]. The flow through the column was gravity-driven. The phenolic fraction was concentrated under reduced pressure (Rotavapor R-210 with vacuum controller V-850, Büchi, Flawil, Switzerland) at a temperature not exceeding 28 °C, and any remaining water was removed via lyophilisation.

### 3.4. LC-MS Analysis

#### 3.4.1. Sample Preparation

The crude/purified extracts (10 mg) were dissolved in a methanol: water solvent (70:30, *v*/*v*). The solutions were filtered through 0.22 µm syringe filters before LC-MS analysis.

#### 3.4.2. RP-UHPLC-ESI-QTOFMS Analysis

Ultra-high-performance liquid chromatography coupled with electrospray ionisation quadrupole time-of-flight mass spectrometry (RP-UHPLC-ESI-QTOFMS) analysis was performed using a Dionex UltiMate 3000 UHPLC (Thermo Fisher Scientific, Sunnyvale, CA, USA) and a Bruker maXis impact ultrahigh-resolution mass spectrometer equipped with an ESI source (Bruker Daltonik, Bremen, Germany). Detection was accomplished in both positive and negative ion modes (ESI+ and ESI−). RP chromatographic separation utilised an Acclaim Polar Advantage II (PA2) column (2.2 µm, 120 Å, 100 × 2.1 mm) from Thermo (Carlsbad, CA, USA). The ESI-MS settings were previously outlined by Mildner-Szkudlarz and co-authors [29]. Molecular ions, [M + H]^+^ and [M − H]^−^, for phenolic compounds were extracted from full-scan chromatograms (±0.005 m/z), and peak areas were integrated using TASQ 2.1 (Bruker Daltonik, Bremen, Germany). The limit of quantification (LOQ, where S/N > 15) for procyanidin B2, epicatechin, gallocatechin, p-coumaric acid, catechin, quercetin, rutin, and 4-hydroxybenzoic acid was determined by injecting a mixture of standards dissolved in methanol:water (7:3, *v*/*v*). The LOQ was not lower than 0.01 µg/mL. Calibration curves were created by injecting standard solutions prepared in the methanol:water (7:3, *v*/*v*) surrogate matrix using an external calibration method. In addition, quality control (QC) samples were prepared and injected before, during, and after the analytical sequence to monitor detector stability and analytical performance throughout the batch analysis. The recovery of standards spiked in samples ranged from 95% to 106%. The coefficient of determination (R^2^) for all calibration curves exceeded 0.99.

#### 3.4.3. Identification of Phenolic Compounds

The compounds in each extract were identified based on the retention time of standards and/or molecular mass, along with structural information obtained from the MS detector during MS/MS experiments.

The tandem mass spectrometric data were used to search for molecular structures using the SIRIUS 6.3.3 software—a Java-based software framework (Friedrich Schiller University, Jena, Germany). SIRIUS integrates CSI:FingerID and CANOPUS (available online: https://bio.informatik.uni-jena.de/software/sirius/ and https://github.com/sirius-ms/sirius/releases?utm_source=chatgpt.com (accessed on 6 May 2025)). The CSI:FingerID is a web service that combines the analysis of isotope patterns in MS spectra with the study of fragmentation patterns in MS/MS spectra to predict molecular fingerprints [30,31,32]. The CANOPUS algorithm was also included to predict compound classes from the molecular fingerprint predicted by CSI:FingerID, without requiring any database search [33]. It is particularly useful to provide structural information when neither spectral nor structural reference data are available. The CANOPUS displays ClassyFire classes [34], which offer a hierarchical chemical classification of small molecules. Meanwhile, the MoNA (MassBank of North America) tandem spectra were downloaded and used for compound annotation in MetaboScape 4.0.4 (Bruker Daltonik, Bremen, Germany) by comparing the assigned MS/MS spectra with the MoNA spectral database.

The results table contains the compound class (computed with CANOPUS) and/or the name of the predicted compound (predicted with CSI:FingerID and/or MoNA). The compound class is sometimes presented only when the results show multiple extremely similar structures with very close fingerprint representations and CSI:FingerID scores. It is noted that the same records appear in the results table, indicating that CSI:FingerID identifies a few similar structures with high scores. Therefore, to avoid overinterpretation, the compounds were conservatively annotated using generalised names such as “Caffeic acid glucoside”, rather than assigning precise structures that could not be reliably confirmed. Some compounds’ MS/MS spectra were recorded in both the positive and negative ion modes. The highest scores were presented, with the MoNA match expressed as a score ranging from 0 to 1000. The accuracy of substructure annotations was indicated by Tanimoto similarity, which computes the percentage similarity of the top-ranked predicted fingerprints among all compounds.

### 3.5. Anti-AGEs Assay In Vitro

#### 3.5.1. BSA-GLU Assay

The inhibition of AGEs formation in the BSA–glucose model was evaluated according to Szawara-Nowak and co-authors [15]. A mixture containing D-glucose (1.0 mol/L), BSA (10 mg/mL), and sodium azide (0.1 mg/mL) in phosphate buffer (0.1 mol/L, pH 7.4) was incubated at 37 °C for 7 days with or without 0.5 mL of the tested extract (0.07–13.31 mg/mL). Fluorescence of AGEs was measured in 96-well black plates at excitation wave (λ_ex_ = 330 nm) and emission wave (λ_em_ = 410 nm) using a FLUOstar Omega (version MARS 3.20) reader (BMG LABTECH, Ortenberg, Germany). Aminoguanidine served as the positive control. All assays were performed in duplicate, and AGEs inhibition (%) was calculated according to the above-cited reference [15]. The IC_50_ values were calculated from linear regression analysis (n = 2, R^2^ = 0.654–0.991).

#### 3.5.2. BSA-MGO Assay

The inhibition of AGE formation in the BSA–MGO model was determined according to Szawara-Nowak and co-authors [15]. A mixture of BSA (1 mg/mL), methylglyoxal (5 × 10^−3^ mol/L), and sodium azide (0.1 mg/mL) in phosphate buffer (0.1 mol/L, pH 7.4) was incubated at 37 °C for 7 days with or without 0.5 mL of the tested extract (0.22–26.62 mg/mL). Fluorescence of AGEs was recorded in 96-well black plates at λ_ex_ = 340 nm and λ_em_ = 420 nm using a FLUOstar Omega reader (BMG LABTECH, Germany). Aminoguanidine served as the positive control. All measurements were performed in duplicate, and AGEs inhibition (%) was calculated following the above-cited reference [15]. The IC_50_ values were calculated from linear regression analysis (n = 2, R^2^ = 0.790–0.999).

#### 3.5.3. BSA-FRU Assay

The inhibition of AGEs formation in the BSA-FRU model was evaluated according to Shen and co-authors [16]. Briefly, fructose (1.5 mol/L) and bovine serum albumin (BSA, 60 mg/mL) were separately dissolved in potassium phosphate buffer (0.2 mol/L, pH 7.4) containing sodium azide (0.6 g/L). Aliquots of BSA solution (0.5 mL) and fructose solution (0.5 mL) were mixed with 0.5 mL of the tested extract (0.02–14.13 mg/mL) at appropriate concentrations. The reaction mixtures were prepared in 10 mL screw-capped test tubes and incubated at 50 °C for 24 h under dark conditions. A control sample was prepared by replacing the extract with deionised water (0.5 mL), while aminoguanidine at corresponding concentrations served as a positive control. After incubation, the fluorescence intensity of the reaction mixtures was measured in 96-well plates at an excitation wavelength (λ_ex_) of 360 nm and an emission wavelength (λ_em_) of 460 nm using a plate reader (SpectraMax iD5, San Jose, CA, USA). The percentage inhibition of AGEs formation was calculated relative to the control sample. All measurements were performed in duplicate. The IC_50_ values were determined by linear regression analysis (n = 2, R^2^ = 0.520–0.917).

### 3.6. Antioxidant Capacity

#### 3.6.1. ABTS Assay

The measurement was performed according to the method described by Horszwald and Andlauer [35]. Briefly, the ABTS•+ solution was diluted with a water: methanol mixture (1:4, *v*/*v*) to an absorbance of 0.70 ± 0.02 at 734 nm. For the spectrophotometric assay, 290 µL of ABTS•+ solution was mixed with 10 µL of sample extract (0.06–5.32 mg/mL), Trolox standard, or blank (water: methanol mixture at the indicated concentration). Absorbance was measured at 734 nm after 6 min incubation at 30 °C. The calibration curve (0–2.0 mM Trolox) was linear (R^2^ = 0.898–1). Results were expressed as IC_50_ values. All analyses were performed in triplicate.

#### 3.6.2. DPPH Assay

The DPPH scavenging activity was determined according to Horszwald and Andlauer [35]. The DPPH• solution was prepared by dissolving 10 mg DPPH• in 250 mL of a water: methanol mixture (1:4, *v*/*v*). For the assay, 300 µL of DPPH• solution was mixed with 20 µL of sample extract (0.06–5.32 mg/mL) or Trolox standard. The mixture was incubated in the dark at room temperature for 30 min, and absorbance was measured at 517 nm using a microplate reader (FLUOstar Omega). The calibration curve (0–2.0 mM Trolox) was linear (R^2^ = 0.929–0.999). Results were expressed as IC50 values. All analyses were performed in triplicate.

### 3.7. Statistical Analysis

Data from AGEs assays are presented as mean ± standard deviation (SD) of duplicate measurements. The differences between samples were analysed by a one-way ANOVA with the Tukey test (*p* < 0.05). The statistical analysis was performed using STATISTICA 13.0 software (StatSoft Inc., Tulsa, OK, USA). Hierarchical clustering dendrograms were generated using all features extracted from MS spectra. The data before clustering were converted into a Z-score by subtracting the mean and dividing by the standard deviation. Ward’s method was used for amalgamation, and Euclidean distances (Dlink) were calculated. The relative ratio (Dlink/Dmax) × 100 was displayed on the vertical axis. The Mann–Whitney U test was used to compare two groups when normality cannot be assessed due to a limited number of samples. Partial Least Squares (PLS) modelling utilised the NIPALS algorithm to analyse non-targeted LC-MS data. Before modelling, variables were standardised with unit-variance scaling to give equal importance to all features. Model performance was assessed through V-fold cross-validation. This method is especially apt for the highly redundant and collinear datasets commonly found in LC-MS metabolomic profiles. Principal Component Analysis (PCA) was conducted to visualise the relationships between the targeted LC-MS data and the samples. These calculations were performed using TIBCO Statistica 14.1.0.4 software (Cloud Software Group, Inc., Fort Lauderdale, FL, USA). The clustered heatmap was generated with R Statistical Software (v 4.4.3; R Core Team [36] using 10.32614/CRAN.package.pheatmap [37].

## 4. Conclusions

This study provides a comprehensive comparative characterisation of polyphenol profiles and bioactivities of *A. arguta* (*cv.* ‘Scarlet September Kiwi’) and *A. kolomikta* (*cv*. ‘Lande’) fruit extracts, clearly demonstrating that both genotype and extract purification significantly determined the chemical composition and functional potential. Using a robust LC-MS/MS approach, 48 polyphenols representing 11 chemical classes were identified, with pronounced qualitative and quantitative differences between the two genotypes. Scarlet fruit extract was distinguished by a higher concentration of total individual polyphenols (TPI) and a profile dominated by quercetin derivatives, whereas Lande extract was characterised by higher relative contributions of caffeic acid derivatives, epicatechin, and procyanidin B2. Purification markedly enhanced the concentration of polyphenols in both genotypes, leading to a substantial increase in TPI values and revealing additional minor constituents. Purified extracts exhibited significantly stronger antiglycation effects across all in vitro models, as well as enhanced antioxidant capacity measured by ABTS and DPPH assays. Multivariate analyses demonstrated that the observed anti-AGEs and antioxidant effects cannot be attributed to single compounds, but rather arise from synergistic interactions within complex polyphenolic matrices. The strong alignment between glycation-inhibitory and antioxidant vectors supports the notion that antioxidant mechanisms play a central role in the modulation of protein glycation by polyphenols.

Despite the promising biological activity of the purified extracts, several limitations of the present study should be acknowledged. The experiments were conducted exclusively using in vitro models, which may not fully reflect the complexity of in vivo physiological conditions and the bioavailability of polyphenolic compounds. Moreover, the relationship between antioxidant activity and protein glycation inhibition was inferred primarily from chemometric and correlation analyses rather than direct mechanistic investigations. Therefore, further studies involving in vivo models and mechanistic approaches are required to confirm the biological relevance and therapeutic potential of the investigated extracts.

## Figures and Tables

**Table 1:** Number of compounds recorded in the crude and purified extracts of *A. arguta* (*cv.* Scarlet September Kiwi) and *A. kolomikta* (*cv.* Lande) across chemical classes.

Classes of Chemical Compounds (ClassyFire, Level 5)	Skarlet		Lande	
	Crude	Purified	Crude	Purified
Flavonoid-O-glycosides	12	16	6	8
Phenolic glycosides	4	5	6	6
Biflavonoids and polyflavonoids	3	8	3	6
Coumaric acids and derivatives	1	4	1	4
Cyclic alcohols and derivatives	2	2	1	2
Catechins	2	2	1	1
Flavan-3-ols	2	2	1	1
Hydroxycinnamic acid glycosides	1	2	1	1
Flavonols	1	1	1	1
Hydroxybenzoic acid derivatives	0	1	0	2
Hydroxyflavonoids	0	1	0	1

**Table 2:** LC-MS analysis of the profile of polyphenol compounds (µg/g dw) in the crude and purified extracts from *A. arguta* (*cv.* Scarlet September Kiwi) and *A. kolomikta* (*cv.* Lande) fruit.

No.	Compounds	Scarlet	Lande	* Lande_1_	Lande_2_	Lande_3_	Scarlet_1_	Scarlet_2_	Scarlet_3_
		Crude Extract		Purified Extract					
**1.**	Salicylic acid glucoside	0.91 ± 0.03	2.98 ± 0.23	8.72 ± 0.05	4.55 ± 0.05	6.13 ± 0.26	2.59 ± 0.12	2.73 ± 0.08	2.83 ± 0.24
**2.**	Caffeic acid glucoside	<LOD	27.16 ± 0.93	123.96 ± 2.06	68.14 ± 0.02	74.38 ± 1.28	<LOD	<LOD	<LOD
**3.**	Quercetin vicianoside	5.96 ± 0.09	<LOD	<LOD	<LOD	<LOD	11.31 ± 0.10	10.71 ± 0.24	12.65 ± 0.23
**4.**	Dihydroxybenzoate glucoside	1.27 ± 0.03	3.55 ± 0.07	11.44 ± 0.65	6.18 ± 0.07	7.22 ± 0.27	3.52 ± 0.01	3.61 ± 0.18	3.34 ± 0.04
**5.**	Gallocatechin	2.33 ± 0.15	<LOD	<LOD	<LOD	<LOD	5.90 ± 0.19	5.84 ± 0.02	6.46 ± 0.08
**6.**	Proanthocyanidin	2.81 ± 0.06	<LOD	<LOD	<LOD	<LOD	7.69 ± 0.6	9.14 ± 0.12	8.99 ± 0.41
**7.**	Flavonoid glycoside (Coumaric acid glycoside)	<LOD	<LOD	<LOD	<LOD	<LOD	3.41 ± 0.01	3.82 ± 0.06	3.34 ± 0.24
**8.**	Coumaric acid glucoside	2.36 ± 0.06	3.71 ± 0.33	13.39 ± 0.08	7.48 ± 0.21	9.22 ± 0.41	6.62 ± 0.60	7.44 ± 0.25	8.13 ± 0.23
**9.**	3.4-dihydroxybenzoic acid	<LOD	<LOD	1.04 ± 0.04	0.66 ± 0.03	0.80 ± 0.01	1.48 ± 0.09	1.40 ± 0.02	1.46 ± 0.02
**10.**	Neochlorogenic acid/Cryptochlorogenic acid	41.90 ± 2.09	<LOD	6.09 ± 0.22	3.51 ± 0.21	4.01 ± 0.10	253.68 ± 13.11	282.84 ± 12.17	303.68 ± 0.05
**11.**	Flavonoid (Taxifolin glucoside)	<LOD	<LOD	1.67 ± 0.08	1.01 ± 0.07	1.23 ± 0.03	4.57 ± 0.22	5.23 ± 0.48	5.57 ± 0.15
**12.**	Fraxin/Isofraxoside	<LOD	2.06 ± 0.17	7.60 ± 0.41	4.29 ± 0.12	5.08 ± 0.11	0.69 ± 0.00	0.76 ± 0.01	0.77 ± 0.01
**13.**	Caffeic acid glucoside	4.02 ± 0.33	294.52 ± 4.78	685.30 ± 10.68	509.16 ± 7.53	544.55 ± 30.13	22.40 ± 0.88	25.42 ± 0.61	28.36 ± 0.34
**14.**	Epigallocatechin	3.75 ± 0.36	<LOD	<LOD	<LOD	<LOD	8.58 ± 0.06	8.39 ± 0.52	9.20 ± 0.01
**15.**	Proanthocyanidin	<LOD	<LOD	<LOD	<LOD	<LOD	8.67 ± 0.60	8.97 ± 0.17	10.53 ± 0.02
**16.**	Sinapoulhexoside	<LOD	8.19 ± 0.00	37.00 ± 0.28	21.01 ± 0.51	24.73 ± 0.22	1.34 ± 0.09	1.42 ± 0.02	2.06 ± 0.01
**17.**	Feruloyl hexoside	6.47 ± 0.36	<LOD	<LOD	<LOD	<LOD	20.69 ± 1.82	24.43 ± 0.16	25.64 ± 0.84
**18.**	Coumaric acid glucoside	<LOD	2.55 ± 0.13	13.42 ± 0.04	6.68 ± 0.00	8.27 ± 0.21	1.93 ± 0.15	1.92 ± 0.02	2.27 ± 0.03
**19.**	Procyanidin	24.19 ± 2.30	15.92 ± 0.60	116.77 ± 1.73	58.26 ± 0.38	72.40 ± 1.60	162.85 ± 2.89	176.34 ± 3.28	187.23 ± 6.93
**20.**	Flavonoid	<LOD	<LOD	1.80 ± 0.00	1.14 ± 0.01	1.29 ± 0.07	2.02 ± 0.12	2.41 ± 0.02	2.33 ± 0.21
**21.**	Feruloylquinic acid	4.63 ± 0.36	<LOD	<LOD	<LOD	<LOD	13.31 ± 0.22	13.95 ± 0.03	15.84 ± 0.01
**22.**	Coumaroyl quinic acid	3.45 ± 0.06	<LOD	1.37 ± 0.00	0.84 ± 0.08	0.81 ± 0.08	10.78 ± 0.76	12.43 ± 0.47	13.16 ± 0.21
**23.**	Catechin	22.07 ± 1.36	12.27 ± 0.96	61.45 ± 0.39	33.99 ± 1.08	40.82 ± 0.19	100.87 ± 8.73	115.96 ± 1.80	121.90 ± 3.82
**24.**	Chlorogenic acid	<LOD	25.60 ± 0.10	51.94 ± 0.02	28.27 ± 0.74	31.99 ± 0.48	<LOD	<LOD	<LOD
**25.**	Epicatechin	24.03 ± 0.24	127.41 ± 1.39	614.42 ± 12.69	384.16 ± 6.07	443.64 ± 8.68	125.74 ± 10.09	145.29 ± 2.10	155.97 ± 1.22
**26.**	Procyanidin B2	29.87 ± 0.82	97.93 ± 7.23	731.23 ± 14.76	413.33 ± 3.46	483.17 ± 0.48	150.92 ± 10.41	169.74 ± 3.86	183.45 ± 4.08
**27.**	Proanthocyanidin	<LOD	<LOD	2.15 ± 0.12	1.48 ± 0.11	1.57 ± 0.14	1.00 ± 0.05	1.04 ± 0.01	1.00 ± 0.06
**28.**	4-hydroxybenzoic acid	<LOD	<LOD	1.02 ± 0.00	0.88 ± 0.01	1.14 ± 0.01	<LOD	<LOD	<LOD
**29.**	Caffeic acid	<LOD	<LOD	0.46 ± 0.00	0.22 ± 0.00	0.28 ± 0.02	0.48 ± 0.01	0.54 ± 0.01	0.61 ± 0.03
**30.**	Quercetin-diglucoside	<LOD	<LOD	<LOD	<LOD	<LOD	7.62 ± 0.71	8.10 ± 0.21	8.68 ± 0.18
**31.**	Flavonoid	<LOD	2.69 ± 0.13	8.45 ± 0.18	5.34 ± 0.09	5.81 ± 0.34	0.81 ± 0.01	0.74 ± 0.06	0.64 ± 0.01
**32.**	Flavonoid glycoside (Myricetin glucopyranoside)	8.40 ± 0.09	<LOD	<LOD	<LOD	<LOD	27.75 ± 2.58	32.61 ± 1.13	33.04 ± 1.10
**33.**	Quercetin glycoside (Quercetin rhamninoside)	3.96 ± 0.03	<LOD	<LOD	<LOD	<LOD	17.75 ± 1.71	20.04 ± 0.25	21.58 ± 0.76
**34.**	Naringenin	<LOD	<LOD	0.29 ± 0.02	0.16 ± 0.00	0.22 ± 0.00	1.46 ± 0.09	1.63 ± 0.11	1.60 ± 0.13
**35.**	Rutin	50.82 ± 1.33	2.79 ± 0.27	11.88 ± 0.23	6.40 ± 0.11	7.66 ± 0.60	317.02 ± 30.15	379.00 ± 5.66	387.86 ± 21.60
**36.**	Proanthocyanidin	<LOD	<LOD	34.91 ± 0.85	17.48 ± 0.01	22.92 ± 1.11	4.97 ± 0.04	5.07 ± 0.25	6.51 ± 0.22
**37.**	Quercetin glucoside	764.76 ± 33.59	52.66 ± 0.56	373.14 ± 7.88	209.84 ± 0.73	243.09 ± 1.57	2198.17 ± 181.46	2411.49 ± 1.67	2386.78 ± 89.64
**38.**	p-Coumaric acid	<LOD	<LOD	1.06 ± 0.02	0.55 ± 0.00	0.78 ± 0.02	0.72 ± 0.01	0.87 ± 0.03	0.85 ± 0.00
**39.**	Flavonoid (Kaempferol glucoside)	9.46 ± 0.45	16.98 ± 0.07	89.95 ± 3.94	45.32 ± 0.15	55.24 ± 1.52	43.08 ± 2.53	37.75 ± 0.04	55.01 ± 2.20
**40.**	Quercetin xyloside	<LOD	<LOD	<LOD	<LOD	<LOD	5.35 ± 0.11	5.79 ± 0.07	5.95 ± 0.16
**41.**	Kaempferol glucoside	10.82 ± 0.63	43.68 ± 2.52	261.74 ± 6.19	142.67 ± 4.26	177.26 ± 0.21	39.87 ± 3.82	45.34 ± 1.19	47.91 ± 2.82
**42.**	Quercetin-3-O-glucosyl-6′-acetate	12.82 ± 0.67	<LOD	<LOD	<LOD	<LOD	54.62 ± 4.20	59.98 ± 2.72	62.66 ± 2.94
**43.**	Quercetin malonylglucoside	19.23 ± 0.91	0.30 ± 0.00	0.85 ± 0.01	0.44 ± 0.01	0.56 ± 0.05	90.34 ± 7.53	98.31 ± 0.51	104.67 ± 4.74
**44.**	Flavonoid glycoside	<LOD	16.48 ± 0.76	53.88 ± 0.90	28.76 ± 1.17	27.93 ± 0.95	<LOD	<LOD	<LOD
**45.**	Flavonoid ferulylglucoside	1.00 ± 0.00	<LOD	<LOD	<LOD	<LOD	4.09 ± 0.08	4.58 ± 0.37	4.88 ± 0.00
**46.**	Flavonoid ferulylglucoside	5.47 ± 0.06	<LOD	<LOD	<LOD	<LOD	16.52 ± 1.09	19.25 ± 0.57	20.92 ± 1.28
**47.**	Flavonoid coumaroyl glycoside	0.60 ± 0.00	<LOD	0.21 ± 0.02	0.11 ± 0.00	0.14 ± 0.01	1.49 ± 0.06	1.81 ± 0.02	1.93 ± 0.02
**48.**	Quercetin	12.15 ± 0.18	1.69 ± 0.13	2.70 ± 0.16	1.65 ± 0.05	1.91 ± 0.08	12.97 ± 0.34	11.70 ± 0.27	14.71 ± 0.81
** TPI		1079.51 ± 46.65	761.13 ± 21.36	3331.33 ± 64.70	2013.98 ± 27.31	2306.21 ± 51.25	3777.62 ± 288.46	4185.83 ± 41.83	4282.94 ± 148.10

The results are expressed as the mean ± SD, n = 2. The Mann–Whitney U test was used to show significant differences in the medians (*p* < 0.05) between the crude and purified extracts for all compounds tested. * Lande1–3 and Scarlet1–3 indicate purified extracts, representing a pooled pair from six independent extract replicates. ** TPI —the sum of individual polyphenol compound concentrations.

**Table 3:** Anti-AGEs and antioxidant potential of the crude and purified extracts from *A. arguta* (*cv.* Scarlet September Kiwi) and *A. kolomikta* (*cv.* Lande) fruit.

Sample	Inhibition (IC_50_) *				
	AGEs			Antioxidant Capacity	
	BSA-GLU	BSA-MGO	BSA-FRU	ABTS	DPPH
*Crude (mg/mL)*					
Scarlet	1.81 ± 0.13 ^Ba^	14.33 ± 0.28 ^Bb^	2.73 ± 0.16 ^Ba^	2.24 ± 0.03 ^D^	5.88 ± 0.20 ^C^
Lande	7.55 ± 0.30 ^Ba^	24.26 ± 0.78 ^Cb^	7.71 ± 0.22 ^Ca^	0.96 ± 0.01 ^C^	1.36 ± 0.04 ^B^
*Purified (mg/mL)*					
Scarlet	0.51 ± 0.03 ^Aa^	0.92 ± 0.00 ^Ab^	0.47 ± 0.00 ^Aa^	0.25 ± 0.01 ^B^	0.67 ± 0.05 ^A^
Lande	0.55 ± 0.01 ^Aa^	1.78 ± 0.00 ^Ac^	0.65 ± 0.00 ^Ab^	0.10 ± 0.00 ^A^	0.26 ± 0.02 ^A^
*Positive control (µg/mL)*					
Aminoguanidine	0.11 ± 0.00	0.11 ± 0.01	0.16 ± 0.02	-	-
Trolox	-	-	-	106.42 ± 0.46	120.29 ± 1.93

The results are expressed as the mean ± SD, n = 2 for anti-AGEs and n = 3 for antioxidant capacity. Capital letters within the same column indicate statistically significant differences between extracts, while lower letters within the same lines indicate statistically significant differences between models of AGEs (one-way ANOVA followed by Tukey’s post hoc test, *p* ≤ 0.05). * IC_50_—the concentrations of fruit extracts (mg/mL) at which AGE formation and radical scavenging are inhibited by 50%.

## Data Availability

The original contributions presented in this study are included in the article/Supplementary Materials. Further inquiries can be directed to the corresponding author.

## Supplementary Materials

The following supporting information can be downloaded at: https://www.mdpi.com/article/10.3390/molecules31111935/s1, Table S1. LC-MS qualitative analysis of polyphenol compounds in the crude and purified extracts from A. arguta (cv. Scarlet September Kiwi) and A. kolomikta (cv. Lande) fruit. Figure S1. Dendrograms created from features derived from MS spectra: A—positive mode (5045), B—negative mode (509). Figure S2. The clustered heatmap was generated using 48 identified from MS spectra. **1**—Salicylic acid glucoside, **2**—caffeic acid glucoside, **3**—quercetin vicianoside, **4**—dihydroxybenzoate glucoside, **5**— gallocatechin, **6**—proanthocyanidin, **7**—flavonoid glycoside (coumaric acid glycoside), **8**—coumaric acid glucoside, **9**—3,4–dihydroxybenzoic acid, **10**—neochlorogenic acid/cryptochlorogenic acid, **11**—flavonoid (taxifolin glucoside), **12**— fraxin/isofraxoside, **13**—caffeic acid glucoside, **14**—epigallocatechin, **15**—proanthocyanidin, **16**—sinapoulhexoside, **17**—feruloyl hexoside, **18**—coumaric acid glucoside, **19**—procyanidin, **20**—flavonoid, **21**—feruloylquinic acid, **22**—coumaroyl quinic acid, **23**—catechin, **24**—chlorogenic acid, **25**—epicatechin, **26**—procyanidin b2, **27**—proanthocyanidin, **28**—4–hydroxybenzoic acid, **29**—caffeic acid, **30**—quercetin–diglucoside, **31**—flavonoid, **32**—flavonoid glycoside (myricetin glucopyranoside), **33**—quercetin glycoside (quercetin rhamninoside), **34**—naringenin, **35**—rutin, **36**—proanthocyanidin, **37**—quercetin glucoside, **38**—p–coumaric acid, **39**—flavonoid (kaempferol glucoside), **40**—quercetin xyloside, **41**—kaempferol glucoside, **42**—quercetin–3–o–glucosyl–6″–acetate, **43**—quercetin malonylglucoside, **44**—flavonoid glycoside, **45**—flavonoid ferulylglucoside, **46**—flavonoid ferulylglucoside, **47**—flavonoid coumaroyl glycoside, **48**—quercetin.
