# Supplementary material for: Purification-Driven Modulation of Polyphenol Profile and Protein Glycation-Inhibitory Potential of Actinidia arguta and Actinidia kolomikta Fruit Extracts
Source: Molecules. 2026 Jun 3;31(11):1935. doi: 10.3390/molecules31111935 (PMC13258577; doi:10.3390/molecules31111935)
Supplement: Supplementary file 1 [file molecules-31-01935-s001.zip › Table S1.pdf]

Table S1. LC-MS qualitative analysis of polyphenol compounds in the crude and purified extracts from *A. arguta* (*cv.* Scarlet September Kiwi) and *A. kolomikta* (*cv.* Lande) fruit.

No.	Compound	Compound formula	Standard used	Rt (min)	Ion	MS/MS	T (%)	CF_5	CF_5
1.	Salicylic acid glucoside	C13H16O8	4-hydroxybenzoic acid	4.22	[M-H]-	863	99	Phenolic glycosides	0.995
2.	Caffeic acid glucoside	C15H18O9	Caffeic acid	4.98	[M-H]-	N/A	82	Phenolic glycosides	0.975
3.	Quercetin vicianoside	C26H28O16	Rutin	5.63	[M+H]+	984	96	Flavonoid-3-O-glycosides	0.979
4.	Dihydroxybenzoate glucoside	C13H16O9	3,4-dihydroxybenzoic acid	5.71	[M-H]-	746	96	Phenolic glycosides	0.994
5.	Gallocatechin	C15H14O7	Gallocatechin	6.03	[M-H]-	959	97	Epigallocatechins	0.976
6.	Proanthocyanidin	C30H26O13	Procyanidin B2	6.05	[M-H]-	N/A	93	Biflavonoids/ polyflavonoids	0.999
7.	Flavonoid glycoside (Coumaric acid glycoside)	C21H20O11	Rutin	6.06	[M-H]-	999	91	Flavonoid O-glycosides	0.917
8.	Coumaric acid glucoside	C15H18O8	p-Coumaric acid	6.06	[M-H]-	996	90	Phenolic glycosides	0.991
9.	3,4-dihydroxybenzoic acid	C7H6O4	3,4-dihydroxybenzoic acid	6.10	[M-H]-	972	98	Hydroxybenzoic acid derivatives	0.998
10.	Neochlorogenic acid/Cryptochlorogenic acid	C16H18O9	Chlorogenic acid	6.18	[M-H]-	999	94	Cyclic alcohols and derivatives	0.999
11.	Flavonoid (Taxifolin glucoside)	C21H22O12	Rutin	6.31	[M-H]-	495	94	Flavonoid O-glycosides	0.900
12.	Fraxin/Isofraxoside	C16H18O10	p-Coumaric acid	6.32	[M-H]-	947	96	Coumarin glycosides	0.998
13.	Caffeic acid glucoside	C15H18O9	Caffeic acid	6.52	[M-H]-	N/A	88	Phenolic glycosides	0.983
14.	Epigallocatechin	C15H14O7	Epigallocatechin	6.63	[M-H]-	985	95	Flavan-3-ols	0.999
15.	Proanthocyanidin	C30H26O13	Procyanidin B2	6.66	[M-H]-	224	95	Biflavonoids/ polyflavonoids	0.990
16.	Sinapoulhexoside	C17H22O10	Sinapic acid	6.71	[M-H]-	N/A	79	Hydroxycinnamic acid glycosides	0.955
17.	Feruloyl hexoside	C16H20O9	Ferulic acid	6.85	[M-H]-	N/A	77	Hydroxycinnamic acid glycosides	0.936
18.	Coumaric acid glucoside	C15H18O8	p-Coumaric acid	6.94	[M-H]-	852	92	Phenolic glycosides	0.964
19.	Procyanidin	C30H26O12	Procyanidin B2	7.01	[M-H]-	909	99	Biflavonoids and polyflavonoids	0.998
20.	Flavonoid	C30H24O12	Procyanidin B2	7.03	[M-H]-	N/A	99	Biflavonoids/ polyflavonoids	0.909
21.	Feruloylquinic acid	C17H20O9	Ferulic acid	7.13	[M-H]-	985	90	Cyclic alcohols and derivatives	0.996
22.	Coumaroyl quinic acid	C16H18O8	p-Coumaric acid	7.14	[M-H]-	961	94	Coumaric acid esters	0.950
23.	Catechin	C15H14O6	Catechin	7.27	[M-H]-	941	97	Flavan-3-ols	0.999
24.	Chlorogenic acid	C16H18O9	Chlorogenic acid	7.37	[M-H]-	994	95	Cyclic alcohols and derivatives	0.999
25.	Epicatechin	C15H14O6	Epicatechin	7.61	[M-H]-	926	97	Catechins	0.999
26.	Procyanidin B2	C30H26O12	Procyanidin B2	7.84	[M-H]-	830	94	Biflavonoids/ polyflavonoids	0.952

27.	Proanthocyanidin	C30H26O11	Procyanidin B2	7.85	[M-H]-	N/A	90	Biflavonoids/ polyflavonoids	0.852
28.	4-hydroxybenzoic acid	C7H6O3	4-hydroxybenzoic acid	8.00	[M-H]-	951	94	Hydroxybenzoic acid derivatives	0.996
29.	Caffeic acid	C9H8O4	Caffeic acid	8.16	[M-H]-	968	94	Coumaric acids and derivatives	0.905
30.	Quercetin-diglucoside	C27H30O17	Rutin	8.20	[M-H]-	987	90	Flavonoid-3-O-glycosides	0.913
31.	Flavonoid	C30H26O11	Procyanidin B2	8.70	[M-H]-	N/A	92	Biflavonoids/ polyflavonoids	0.950
32.	Flavonoid glycoside (Myricetin glucopyranoside)	C21H20O13	Rutin	8.82	[M-H]-	994	87	Flavonoid-3-O-glycosides	0.682
33.	Quercetin glycoside (Quercetin rhamnoside)	C33H40O20	Rutin	8.83	[M-H]-	995	97	Flavonoid O-glycosides	0.984
34.	Naringenin	C15H12O5	Apigenin	8.90	[M+H]+	964	97	4'-hydroxyflavonoids	0.865
35.	Rutin	C27H30O16	Rutin	9.01	[M-H]-	997	98	Flavonoid O-glycosides	0.993
36.	Proanthocyanidin	C30H26O12	Procyanidin B2	9.11	[M-H]-	762	94	Biflavonoids/ polyflavonoids	0.942
37.	Quercetin glucoside	C21H20O12	Rutin	9.51	[M-H]-	990	98	Flavonoid-3-O-glycosides	0.989
38.	p-Coumaric acid	C9H8O3	p-Coumaric acid	9.75	[M-H]-	981	92	Coumaric acids	0.987
39.	Flavonoid (Kaempferol glucoside)	C21H20O11	Rutin	9.92	[M-H]-	1000	95	Flavonoid-O-glycosides	0.593
40.	Quercetin xyloside	C20H18O11	Rutin	10.01	[M-H]-	924	96	Flavonoid O-glycosides	0.996
41.	Kaempferol glucoside	C21H20O11	Rutin	10.09	[M-H]-	992	98	Flavonoid O-glycosides	0.956
42.	Quercetin-3-O-glucosyl-6"-acetate	C23H22O13	Rutin	10.30	[M-H]-	984	98	Flavonoid-3-O-glycosides	0.986
43.	Quercetin malonylglucoside	C24H22O15	Rutin	10.30	[M-H]-	976	97	Flavonoid O-glycosides	0.996
44.	Flavonoid glycoside	C38H46O21	Rutin	11.01	[M+H]+	N/A	97	Flavonoid O-glycosides	0.976
45.	Flavonoid ferulylglucoside	C31H28O15	Ferulic acid	11.55	[M-H]-	N/A	99	Flavonoid-3-O-glycosides	0.959
46.	Flavonoid ferulylglucoside	C31H28O15	Rutin	11.68	[M-H]-	N/A	99	Flavonoid-3-O-glycosides	0.957
47.	Flavonoid coumaroyl glycoside	C30H26O14	p-Coumaric acid	12.04	[M-H]-	N/A	97	Flavonoid O-p-coumaroyl glycosides	0.549
48.	Quercetin	C15H10O7	Quercetin	13.81	[M-H]-	957	99	Flavonols	0.926
