# Supplementary figures and images for: Purification-Driven Modulation of Polyphenol Profile and Protein Glycation-Inhibitory Potential of Actinidia arguta and Actinidia kolomikta Fruit Extracts
Source: Molecules. 2026 Jun 3;31(11):1935. doi: 10.3390/molecules31111935 (PMC13258577; doi:10.3390/molecules31111935)

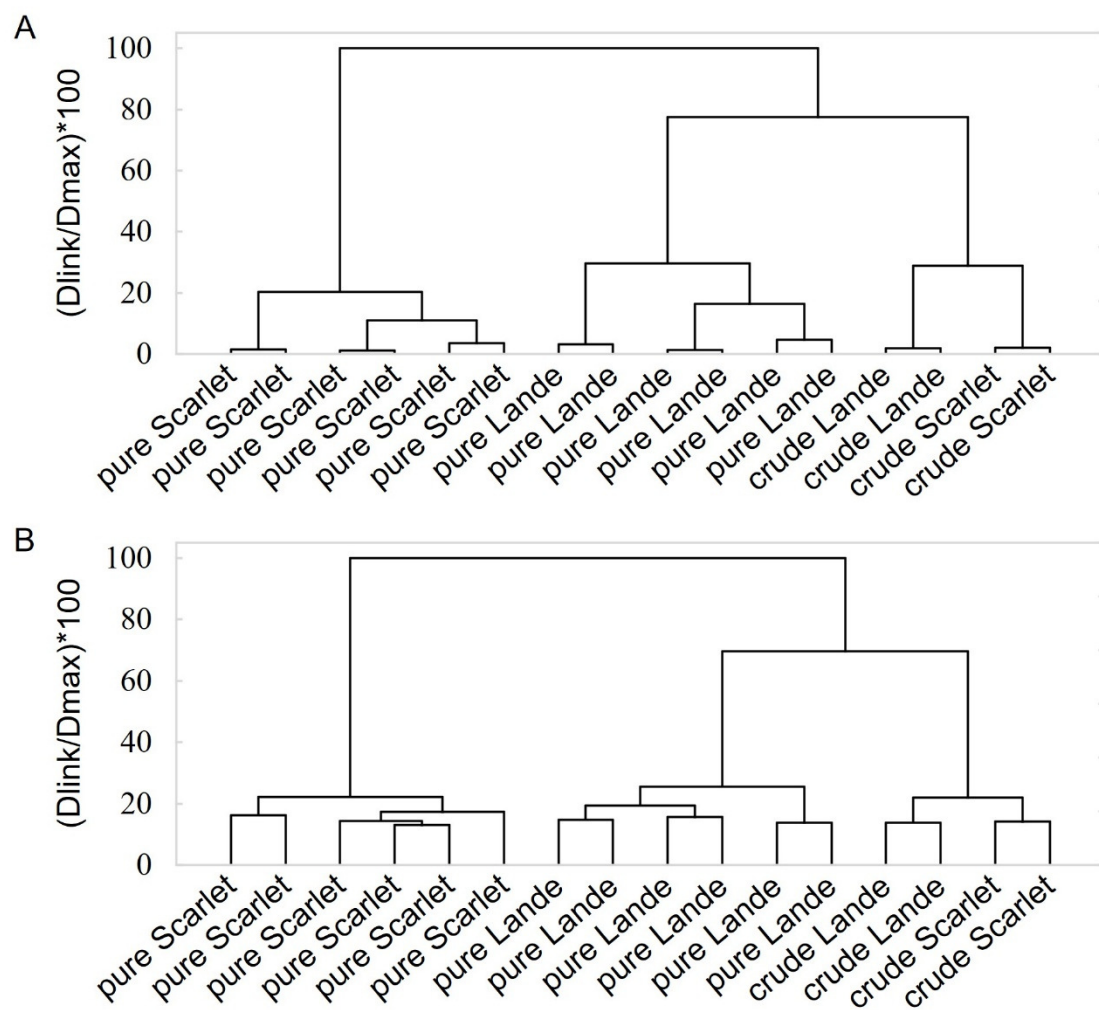

Figure S1

Supplement: Supplementary file 1 [file molecules-31-01935-s001.zip › Figure S1.pdf]

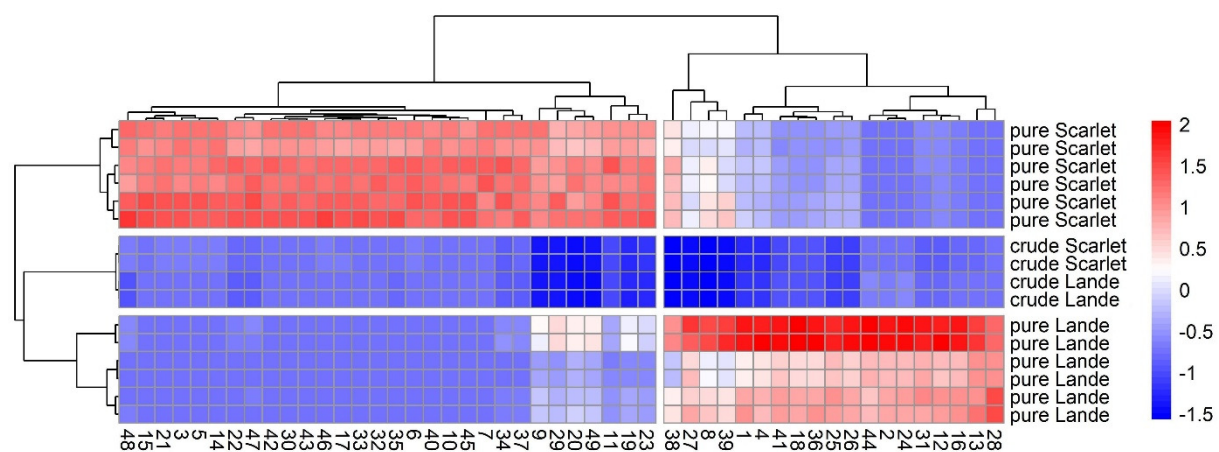

Figure S2

Supplement: Supplementary file 1 [file molecules-31-01935-s001.zip › Figure S2.pdf]
